# Remote Support of Elderly Women Participating in Mental Health Promotion Programme during the COVID-19 Pandemic: A Single-Group Longitudinal Intervention

**DOI:** 10.3390/ijerph19074073

**Published:** 2022-03-29

**Authors:** Karolina Juszko, Anna Serweta, Błażej Cieślik, Wojciech Idzikowski, Joanna Szczepańska-Gieracha, Robert Gajda

**Affiliations:** 1 Faculty of Physiotherapy, Wroclaw University of Health and Sport Sciences, 51-612 Wroclaw, Poland; serwetanna@gmail.com (A.S.); joanna.szczepanska@awf.wroc.pl (J.S.-G.); 2 Faculty of Health Sciences, Jan Dlugosz University, 42-200 Czestochowa, Poland; blaze.cieslik@gmail.com; 3 Faculty of Physical Education and Sport Sciences, Wroclaw University of Health and Sport Sciences, 51-612 Wroclaw, Poland; wojciechidzikowski@gmail.com; 4 Gajda-Med District Hospital, 06-100 Pultusk, Poland; gajda@gajdamed.pl; 5 Department of Kinesiology and Health Prevention, Jan Dlugosz University, 42-200 Czestochowa, Poland

**Keywords:** social isolation, mental health, remote support, depression, women, elderly

## Abstract

The aim of the study was to evaluate the longitudinal changes in mental well-being during the switch of the intervention from a personal contact to a remotely delivered Mental Health Prevention and Promotion Programme in elderly women at high risk of developing depression. The study included 70 women aged over 60 with a mean age of 72.28 years. In order to determine mental well-being, the Geriatric Depression Scale (GDS) was used at four time points: January, April, September and December 2020. A self-developed questionnaire was used to determine predictors of mental well-being. Two-way ANOVA demonstrated a lack of significant differences between the means in the GDS scores at the four data collection time points (*p* = 0.21). Frequent use of green areas reduced the GDS score on average by 1.52 points (*p* = 0.01), while owning a garden by 1.51 points (*p* = 0.04). The illness of a family member increased the GDS score by an average of 1.7 points (*p* = 0.02). No significant mood deterioration was found between January 2020 and December 2020 in the studied group of elderly women at a high risk of developing depression, which suggests that the remote support provided in the mental health promotion programme was effective.

## 1. Introduction

In December 2019, a case of a new disease caused by the SARS-CoV-2 virus was detected for the first time in Wuhan, China [1]. The world has been overrun by the rapidly spreading COVID-19, which has significantly changed the priorities of public health. The pandemic has made it necessary to mobilise all the available health units to care for those infected and to prevent the spread of the disease. The first confirmed case of the infection in Poland was reported on 4 March 2020 [2]. Since 13 March 2020, restrictions have been maintained continuously to prevent the transmission of the virus.

Most concerns have been raised by the situation of the elderly, as this group has the highest rate of COVID-19-related complications, and also of those resulting in death [3]. Therefore, during the early period of the pandemic, in Poland senior citizens were subject to the greatest restrictions, including a ban on leaving their homes [4]. All health promotion programmes for the elderly realised in personal contact were stopped overnight. These programmes guaranteed seniors at risk of social exclusion not only regular contact with other people in similar life circumstances but also support from therapists.

A prolonged lockdown, which lasted almost the entire year of 2020, caused chronic stress that directly affected the mood and levels of perceived anxiety in the population at large [5]. We assume that the severity of mood disorders during the pandemic may have been particularly pronounced in individuals who had already been at high risk prior to the pandemic and had been receiving therapeutic support aimed at preventing social exclusion [6]. The continuation of this support appears to be extremely important, as psychological distress related to social isolation could significantly affect the mental state of this group of people [7,8]. Unfortunately, so far, there are no studies evaluating the effectiveness of remotely-delivered interventions concerning loneliness and psychological distress in older adults during the current COVID-19 pandemic [9].

Therefore, the main aim of the study was to evaluate the longitudinal changes in mental well-being during the switch of the intervention to a remotely delivered Mental Health Prevention and Promotion Programme in elderly women at high risk of developing depression. The additional aim was to identify the factors associated with the well-being of its participants (protective factors) and the factors associated with greater severity of depressive symptoms in the specific situation of a pandemic (predictors of depression). We developed three different models for factors promoting better well-being.

The ‘Environmental’ model took into account the fact of owning a garden, the frequency of using green areas and of taking part in physical activity. The ‘Social’ model took into account marital status, housing circumstances (presence and number of co-habitants) and maintenance of contact with one’s family and friends. The ‘Covid’ model was related strictly to the pandemic situation and took into account the occurrence of the COVID-19 disease in a family member or a friend. Based on the above models, we assumed the following hypotheses: (1) having one’s own garden or frequent use of green areas protects against the development of depression during the COVID-19 pandemic, (2) living together with a husband and/or children or frequent contact with family and friends protects against the development of depression during the COVID-19 pandemic, and (3) Covid infection detected in a family member or friend increases the risk of developing depression during the COVID-19 pandemic.

## 2. Materials and Methods

### 2.1. Participants and Study Setting

The study design was set as a single-group longitudinal intervention. The study included 70 women aged over 60 with a mean age of 72.28 ± 5.22 years. All women were participants in the Mental Health Prevention and Promotion Programme run by the Foundation for Senior Citizen Activation, ‘Siwy Dym’, in Wroclaw, Poland. The project has been run continuously since 2016 and starts a new edition every year [10,11,12] thanks to funding provided by the Health Department of the Municipality of Wroclaw, Poland. The programme is aimed at women over 60 years of age who, due to their life or health circumstances, are at risk of social exclusion and were diagnosed with depression before. Regular meetings of a support group (10–12 people), incorporating general physical exercises and relaxation sessions, health education and psycho-education play a key role in the project.

The assumptions of the described model are in accordance with the newest recommendations [13], and its effectiveness has been empirically supported [10,11,12]. New female participants are admitted to the programme at the beginning of each calendar year, but the majority of the group are those who continue their participation from year to year. Inclusion criteria consist of a physician’s consent and an up-to-date health examination, together with the results of laboratory blood tests, including fasting glucose, total cholesterol, LDL, HDL and triglyceride levels. In 2020, regular classes started on 15 January and ran according to the project schedule until 15 March 2020, when, pursuant to the recommendations of the Polish Chief Sanitary Inspector, the activities were suspended due to their group character and the risk of spreading the coronavirus.

Finally, 68 of the included women completed the study. Most of the project participants had secondary education (43.7%), the second-highest percentage had higher education (29.6%), about 10% had vocational education, while 16.9% did not provide an answer to the question regarding their education level. Nearly 40% of the study group were married, slightly more than 30% were widowed, about 14% were single and 4% of the women provided no answer regarding their marital status. Those living alone accounted for 46.5% of the group, those living with a husband nearly 30%, with children 8.4% and 11% declared that they lived together with a husband and a child/children, while 6% of women did not provide an answer to the marital status question.

The type of physical activity most often undertaken by the respondents was exercising at home (approx. 48%), outdoor activities accounted for nearly 45% (walking 32% and gardening 13%) of the reported physical activities, one participant declared that she did not engage in any physical activities and 6% of women did not provide an answer to the physical activity question. Almost half of the women (48%) declared that they did not undertake regular physical exercise, and a similar number (47%) declared that they undertook regular physical activity. Four respondents did not provide an answer to this question. Of the respondents, 36 exercised nearly every day (over 50%), 19 respondents (approx. 27%) exercised twice a week, 12 respondents (17%) performed hardly any exercise and 4 respondents provided no answer to this question. Selected clinical parameters in the study group are presented in Table 1.

### 2.2. Remote Support Programme

Since 1 April 2020, support within the project was delivered remotely. The Multimodal Mental Health Prevention and Promotion Programme comprised three elements: (1) maintaining previously established relationship through telephone contact, (2) providing general fitness exercises guidelines for in-home execution and recording of a relaxation session for daily listening and (3) providing professional psychotherapeutic care if the participant’s mental state deteriorates.

The relationship established with female participants in previous months of the project was now maintained through telephone contact. If such a need arose, food, medicine, books and protective masks were delivered to the participants through personal contact, and other urgent daily life matters were taken care of on their behalf. However, all the participants were physically fit enough to attend to their own basic matters of daily life. It quickly became apparent that the need for support was related primarily to maintaining personal relationships, reducing loneliness in a lockdown situation and reducing separation anxiety.

Each participant was telephoned once a week and the conversation lasted between 10 and 20 min, depending on her needs. Moreover, all the participants were sent a relaxation session during the lockdown designed particularly for this age group. The foundation running the programme also made the recorded session available on its Facebook page, YouTube channel or provided via the WhatsApp mobile application. The person in constant contact with the project participants reminded them to listen regularly to the recording. In addition, each participant was provided with a handout of the general fitness exercises they had been doing in group classes prior to the outbreak of the pandemic and they were encouraged to train regularly at home.

**Figure 1:**
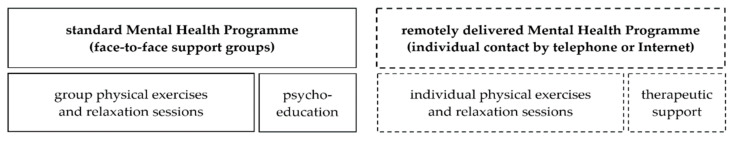
Standard support program and a remotely delivered support program.

During the course of the project (January–December 2020), the participants’ sense of well-being and mood levels were measured four times using the Geriatric Depression Scale (GDS), in January, April, September and December. Participants who showed high levels of depressive symptoms received individual support from a psychotherapist; the conversation then lasted about 50 min and had the character of a crisis intervention rather than a psychotherapy session. The switch of the intervention to a remotely delivered one is presented in Figure 1.

### 2.3. Outcome Measures

The 30-item GDS was used to assess mental health. It is a self-rating screening tool to measure depression symptoms in older adults. It contains 30 ‘yes’ or ‘no’ items. A result between 0 and 10 points indicates a lack of depression, while a score of 11 or above indicates depression of increasing severity. The scale provides high reliability (Cronbach’s *α* = 0.69–0.99) and validity [14]. The initial assessment of well-being and mood using the GDS was carried out just before the outbreak of the pandemic (January 2020) and was meant to constitute the baseline measurement for the later evaluation of the efficacy of the programme. Therefore, the assessment was carried out four times: in January, April, September and December.

Additionally, a self-developed questionnaire was administered and used to create three models: ‘Environmental’, ‘Social’, and ‘Covid’. The ‘Environmental’ model included questions regarding access to one’s own garden (yes/no), frequency of using green areas (often/sometimes/rarely) and regular physical activity (yes/no). The ‘Social’ model included questions regarding marital status (alone/with spouse/with child/with husband and child), type of contact with family (physical/telephone/internet), frequency of maintaining contact with one’s family and friends (almost daily/at least once a week/once every two weeks or less) and way of travelling (car/public transport/walking). The ‘Covid’ model included questions on diseases of relatives (yes/no) and diseases of friends (yes/no). This questionnaire was administered in December 2020. Both of the outcome measures were carried out by an independent outcome assessor.

### 2.4. Data Analysis

The obtained data were analysed with the IBM Statistical Package for the Social Sciences 26.0 software (IBM Corp., Armonk, NY, USA). The research material was analysed using descriptive statistics, including mean, median, standard deviation and percentages. Prior to analysis, the data distribution was tested for normality using the Shapiro–Wilk test. A two-way repeated analysis of variance (ANOVA) was used to examine the difference at each time point and the relationship between owning a garden and the GDS levels (with all the measurement points included). Multiple linear regression (stepwise) was used to identify the association between the variables extracted in the aforementioned models and the participants’ well-being and mood as expressed by the GDS scale (during the pandemic period, i.e., April, September and December). Prior to the multiple linear regression analysis, the assumption of a linear relationship (using Pearson correlation coefficient) between the outcome variable and the independent variables was tested. A significance level of α < 0.05 was established.

## 3. Results

### 3.1. Mental Well-Being

**Figure 2:**
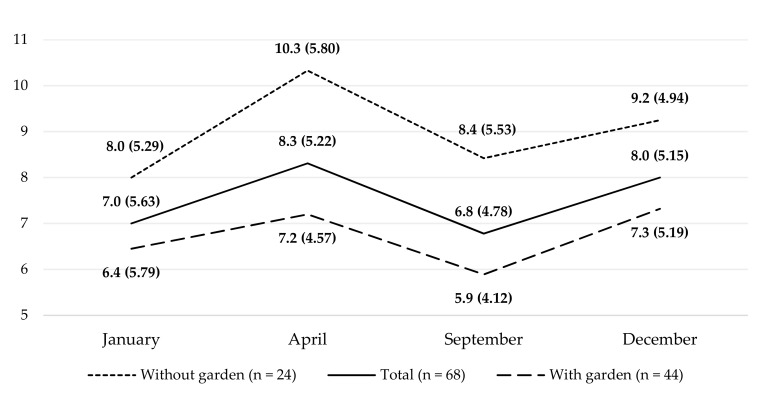
Intensification of depression symptoms at subsequent measurement points in the entire study group and groups with or without access to a garden.

The mean GDS score obtained by the women enrolled in the project (from January to December 2020) was 7.7 (±4.63) points. Although depression is highly prevalent in older adults and associated with increasing age, the intervention effect did not significantly differ between younger old (aged < 75) and older old (75+), therefore the research group was not divided due to age. Two-way ANOVA demonstrated a lack of significant differences between the means in the GDS scores at the four data collection time points (*p* = 0.21, Table 2). The distribution of mean GDS scores at the individual measuring points is presented in Figure 2. Intensification of depressive symptoms was not related to age, education or the measured health parameters (BMI, total cholesterol, LDL, HDL, triglyceride levels, fasting glucose and blood pressure).

The relationship between owning a garden and GDS levels (with all measurement points included) was examined using a multi-factor ANOVA, which showed that access to one’s own green space was significantly associated with better well-being among the female seniors throughout the period of the study. Participants owning a garden demonstrated, both before the pandemic and throughout the study period, better well-being than the mean for all the participants and participants without access to a garden. The two-way ANOVA results are presented in Table 2 (were included only the significant results).

### 3.2. Predictors of Better Well-Being

The multiple linear regression results for the ‘Covid’ model with GDS are presented in Table 3 (only the significant results were included). As regards the models developed for factors promoting better well-being, no statistically significant relationships were found between the ‘Social’ model (which included factors such as living alone or with a member of the family, marital status and maintaining contact with one’s family and friends) and the level of well-being and mood of the participants. Although 21% of the women maintained personal contact with their families and 76% maintained contact only by telephone, this had no significant relationship with the obtained GDS scores. Likewise, contact with friends, whether daily (as was the case in 75% of the group) or at least once a week (in 21% of the group) was not found to be a significant predictor of good well-being.

Analysis of the ‘Covid’ model demonstrated that an incidence of the coronavirus disease among friends did not significantly affect the participants’ levels of well-being and mood, whereas the disease in a family member did. The illness of a family member increased the GDS score by an average of 1.7 points (*p* = 0.02). In the ‘Environmental’ model taking into account the frequency of visiting green areas, ownership of a garden by a participant or her immediate family and being physically active, the last variable was excluded at the analysis stage. The remaining variables related to contact with nature could be a significant predictor of good well-being. Frequent use of green areas reduced the GDS score on average by 1.52 points (*p* = 0.01), while owning a garden reduced it by 1.51 points (*p* = 0.04).

## 4. Discussion

### 4.1. This Study

This study had two objectives; the main aim of the study was to evaluate the impact of a remotely delivered multimodal Mental Health Prevention and Promotion Programme in elderly women at high risk of developing depression. The secondary objective was to identify the factors associated with the well-being of the female participants of the study. Regarding the main aim of the study, the statistical analysis demonstrated that the mean GDS score did not differ significantly between the measured time points (January, April, September and December 2020). In other words, over the whole year, there was no mood deterioration in a studied group. The GDS mean score for January 2020 differed by only one point from the score for December 2020 (7.0 vs. 8.0), when the epidemic situation in Poland was terrible. At this point, it is important to emphasise that this was a group at high risk of developing depression due to previous psychiatric problems. In this study, there was no control group without a support programme. All the women who were under the care of the Foundation for Senior Citizen Activation at that time received the remote support. It would have been unethical to omit anyone in order to create a control group. Therefore, the results should be interpreted with caution.

However, despite the lack of a control group, the above results should be viewed in the context of the participants’ situation. Among older adults, social isolation is associated with increased reactivity to stressors, anxiety and depression [9,15]. A recent study demonstrated that social disconnection puts the elderly at greater risk of depression [16]. This risk appears to be higher in people who have been diagnosed with depression in earlier years [17]. The lockdown period and associated social isolation had a statistically significant effect on depression and anxiety levels during the early COVID-19 pandemic in the elderly population [7]. The same study demonstrated that women, compared to men, were more likely to report worsened components of anxiety and depression [7,18]. In light of these results, we can speculate that our remote support may have supported the lack of mood deterioration observed in our study group. It is worth noting that the support was delivered by people who had previously provided therapeutic activities in the same group. Thus, stable therapeutic relationships had already been established prior to the outbreak of the pandemic and were effectively maintained during its course. Nevertheless, given the lack of a control group, further research is necessary.

Available research confirmed the need to provide large-scale care for the mental health of specific groups within society while taking into account the constraints of the sanitary and epidemiological regime [19]. Ravindran et al. (2020) described their experience of providing psychological support through a national telephone helpline [20]. In doing so, they observed that the helpline was only an initial step and that other remote support actions were necessary on a larger scale. Similar conclusions were reached in the study by Reilly et al. (2020), who additionally observed better outcomes for patients using virtual rather than telephone-based consultations [21]. According to Solomou and Constantinidou (2020), during a pandemic, female gender is a predisposing factor for the development of depressive and anxiety symptoms, so women should be the first to receive help [22]. The authors’ observations show that maintaining relationships between people, even over the telephone, is extremely important. Research findings indicate the presence of significant associations between the gradual formation and maintenance of relationships and the participants’ relatively stable levels of well-being and mood, despite their previous psychiatric problems and the fact that they were experiencing a difficult, unprecedented situation of a pandemic and were subject to a lockdown [23,24].

A special mention must be made of the results related to the role of gardens and green areas in preserving good mental health. All the participants lived in a large city with a population of over 600,000, where dense development of the central districts makes contact with nature impossible for many inhabitants, even if only in the form of a nice view out of the window. Perhaps this is why not owning a garden or not being able to enjoy green spaces was so distressing for the participants and had a significant impact on their well-being. As shown in a study by Nishigaki et al. (2020), in urban environments, there is a correlation between areas with more trees and a lower likelihood of depression [25]. This should be a very important guideline for urban planners and those involved in shaping the urban landscape. At the same time, we were surprised by the results showing how little impact the living situation had on the mental state of the senior women. This refers to marital status or the sharing of a household with other family members. Many years of therapeutic practice confirm these results, clearly showing that it is actually the quality of a relationship that is more important than the mere fact of having children or a spouse [26].

### 4.2. Strengths and Limitations

This study provides valuable insights on several levels. One of these was certainly a longitudinal measurement of changes in mood over a one-year period. In addition, the first mental state assessment was made just before the first case of SARS-CoV-2 appeared in Poland, and subsequent measurements were made in April, September and December 2020 when the epidemic situation in Poland was changing very dynamically. Moreover, the study included a group of elderly women who had an episode of depression in the past, making them a group at high risk of developing depression during the pandemic.

Furthermore, the unique multimodal method in which remote support was conducted during the pandemic can be considered a strength. Finally, the evaluation of the predictors within the three models provides valuable insight into the risk factors for depression in regard to diseases in the family and the lack of green environment available.

Nevertheless, the limitations of the study should be outlined. Firstly, the lack of a control group greatly limits the interpretation of the results. Secondly, due to the unexpected outbreak of the pandemic, the nature of the research was abruptly changed and, for instance, it was not possible to include measurements of the number of steps taken per day or the average daily pulse. Such information could be an interesting addition to the study, complementing it with more objective measurements of physical activity. Therefore, the results of the study should be interpreted with caution.

### 4.3. Future Research Direction

In the future, the authors plan to extend the methodology to investigate in even greater detail the impact of the above-mentioned models on the well-being and mood of women aged 60 plus, in the post-COVID period, as well.

## 5. Conclusions

In conclusion, no significant mood deterioration was found between the subsequent measurements of well-being between January 2020 and December 2020. This means that the multimodal remote support was effective, even in such a difficult, unprecedented situation. However, due to the lack of a control group, these results should be interpreted with caution. Taking into account the measurements from all time points, having one’s own garden or use of green areas were significant predictors of better well-being during the pandemic. It follows that contact with nature is extremely important and, when introducing restrictions, the use of publicly accessible green areas should never be prohibited, but it should only be ensured that such areas may be used safely. On the other hand, the COVID-19 illness of a family member is a predictor of a greater mood deterioration in an elderly person. In such a situation, remote psychological support should be provided as soon as possible.

## Figures and Tables

**Table 1:** Participants’ baseline characteristics.

Variable	Mean (SD)	Total Range
n	68	–
Age (years)	72.28 (5.22)	62–86
Body mass (kg)	74.66 (14.00)	43.00–110.00
Height (m)	1.60 (0.06)	1.47–1.73
BMI ^1^ (kg/cm^2^)	28.17 (6.20)	18.01–39.67
Total cholesterol (mg/dL)	214.21 (41.44)	143.90–342.00
HDL-C ^2^ (mg/dL)	73.04 (30.18)	36.00–183.00
LDL-C ^3^ (mg/dL)	119.58 (40.03)	51.00–238.00
Triglycerides (mg/dL)	128.78 (51.40)	52.00–293.00
Fasting glucose (mg/dL)	97.92 (20.01)	60.00–147.30
Resting SBP ^4^ (mm Hg)	131.33 (18.11)	90.00–184.00
Resting DBP ^5^ (mm Hg)	73.78 (8.32)	56.00–88.00

^1^ BMI: Body Mass Index; ^2^ HDL-C: High-Density Lipoprotein Cholesterol; ^3^ LDL-C: Low-Density Lipoprotein Cholesterol; ^4^ SBP: Systolic Blood Pressure; ^5^ DBP: Diastolic Blood Pressure

**Table 2:** Two-way ANOVA results (GDS for each measuring point).

Variable		Mean Square	*F*	ηp^2^	*p*
GDS ^1^	Time	39.51	1.51	0.02	0.21
	Garden	324.07	12.38	0.04	0.001
	Time * Garden	7.47	0.29	0.00	0.83

^1^ GDS: Geriatric Depression Scale. * means the interaction between “Time” and the “Garden”.

**Table 3:** Multiple linear regression results for the ‘Covid’ and ‘Environmental’ models with GDS during the pandemic (April, September and December).

Models	*β*	Standardised *β*	Adjusted R^2^	*t*	95% CI ^1^	*p*
‘Environmental’ model						
Green areas	−1.52	−0.18	0.05	−2.54	−2.71 to −0.34	0.01
Garden	−1.51	−0.15	0.06	−2.02	−2.99 to −0.04	0.04
‘Covid’ model						
Disease in the family	1.70	0.17	0.02	2.34	0.27 to 3.13	0.02

^1^ CI: Confidence Interval.

## Data Availability

The data presented in this study are available on request from the corresponding author. The data are not publicly available due to privacy restrictions.
